# Topography of Movement-Related Delta and Theta Brain Oscillations

**DOI:** 10.1007/s10548-021-00854-0

**Published:** 2021-06-15

**Authors:** János Körmendi, Eszter Ferentzi, Béla Weiss, Zoltán Nagy

**Affiliations:** 1 Faculty of Information Technology, Department of Electrical Engineering and Information Systems, University of Pannonia, Egyetem utca 2, 8200 Veszprém, Hungary; 2 Institute of Health Promotion and Sport Sciences, ELTE Eötvös Loránd University, Bogdánfy Ödön u. 10/B, 1117 Budapest, Hungary; 3 National Institute of Clinical Neurosciences, Amerikai út 57, 1145 Budapest, Hungary; 4 Semmelweis University, Üllői út 26, 1085 Budapest, Hungary; 5 Brain Imaging Centre, Research Centre for Natural Sciences, Magyar tudósok körútja 2, 1117 Budapest, Hungary

**Keywords:** EEG power dynamics, Finger tapping, Scalp current density, Event-related synchronization, Delta oscillations, Theta oscillations

## Abstract

The aim of this study was to analyse the high density EEG during movement execution guided by visual attention to reveal the detailed topographic distributions of delta and theta oscillations. Twenty right-handed young subjects performed a finger tapping task, paced by a continuously transited repeating visual stimuli. Baseline corrected power of scalp current density transformed EEG was statistically assessed with cluster-based permutation testing. Delta and theta activities revealed differences in their spatial properties at the time of finger tapping execution. Theta synchronization showed a contralateral double activation in the parietal and fronto-central regions, while delta activity appeared in the central contralateral channels. Differences in the spatiotemporal topography between delta and theta activity in the course of movement execution were identified on high density EEG.

## Introduction

The relationship between voluntary movements and the corresponding bioelectric activities reflects fundamental mechanisms of cortical functions (Harmony 2013; Klimesch et al. 2007; Pfurtscheller and Lopes da Silva 1999). Event-related potentials (ERPs), i.e., the averaged amplitude fluctuations triggered to the repetitions of well-defined trials had been described in the classical EEG literature (Deecke et al. 1969; Kristeva et al. 1979). ERP potentials evoked by execution of voluntary movements are occasionally called movement-related cortical potentials (Hallett 1994), or movement-related potentials (MRP) (Georgiev et al. 2016). Various paradigms and methods were used to initiate movements (Brunia et al. 2012). Basically two types of motion-induction are widely applied, i.e., self-induced voluntary tapping (Brunia et al. 2012) where the EEG data are triggered to the movement onset, and Go/NoGo paradigms (Kirmizi-Alsan et al. 2006) where the triggering event is the onset of the Go/NoGo stimulus.

The ERP can be regarded as a superposition of oscillations with different amplitude and phase aligned to the event (Pfurtscheller and Lopes da Silva 1999). To examine the detailed bioelectric characteristics of the oscillations, frequency decomposition separates the parallel activities superimposed in a well-defined time domain (Kirmizi-Alsan et al. 2006). The most studied movement-related oscillatory phenomenon is the event-related desynchronization (ERD), the amplitude decrease in alpha and beta waves during preparation and execution of motor activity (Gastaut 1952; Pfurtscheller 1981; Pfurtscheller and Aranibar 1979).

In the case of Go/NoGo experimental paradigms, decision making is related to theta and delta synchronization in frontal and central areas. These phenomena are regarded as EEG signs of sustained attention or response inhibition (Kirmizi-Alsan et al. 2006; Yamanaka and Yamamoto 2010). In voluntary movements, delta and theta oscillations were also described; these frequency bands however, were not documented separately, and the source of these activities was also debated (Popivanov et al. 1999; Popovych et al. 2016). These oscillations were either localized in the contralateral primary motor cortex (Popovych et al. 2016) or in the supplementary motor area (Popivanov et al. 1999). Medial prefrontal-frontal theta activity is a compelling candidate by which cognitive control may be realized (Cavanagh and Frank 2014).

In this study, we used a simple paradigm in which motor execution is guided by visual attention. The aims were twofold. Firstly, to describe separately the delta and the theta synchronizations. Secondly, to localize the topolographic extension in the spatial- temporal-spectral domains of the these frequency bands.

## Methods

### Participants

Twenty young right-handed subjects (10 male) with a mean age of 23.6 years (standard deviation, SD = 2.9 years; range 18–30 years) participated in the study. None of them had any history of neurological or psychiatric diseases and they had normal or corrected-to-normal vision. Handedness of participants was assessed with the Edinburgh Handedness Inventory (EHI) score (Oldfield 1971). The average EHI score was 81.9 (SD = 14.1; range: 67–100). The study was approved by the local ethics committee of the National Institute of Clinical Neurosciences, Budapest. The experiments followed the approved protocol. Subjects signed an informed consent form before participation.

### Experimental Procedure

The experimental used in this study was developed to meet two major requirements. One was to create a condition close to a realistic situation, where motor action is cued by a visual stimulus or event, but the timing of index finger tapping was connected to the decision of brightness or darkness on the screen figure. The classical paradigms like the ones involving voluntary movement without any external stimuli and the Go/NoGo tasks with focused attention have relatively low ecological validity. In real life, situations are common in which a person starts a voluntary movement after a more or less predictable visual cue. The other requirement of the paradigm was the simplicity of the task since its future application in clinical studies is also planned with patients with motor function disorders, such as stroke with mild upper limb paresis or Parkinson disease.

The EEG experiment started with closed- and open-eyes resting-state recordings (3 min each), followed by the finger tapping task. Finger tapping was cued by a visual stimulus that was presented on a 22" Samsung 2253BW display with a resolution of 1680 × 1050 pixels. The visual stimulus consisted of a small square (side length of about 0.8 visual degrees) on a middle grey background. The square was positioned on the centre of the screen and its colour changed regularly and gradually from middle grey to black and back with a periodicity of 10 s. Subjects sat in front of the screen at a distance of about 70 cm. They were instructed to press a push-button on a custom-made feedback panel with their index finger when the contrast between the square and the background was minimal or maximal (response stimuli). Finger tapping was performed 150 times by the left and right index fingers in two separate blocks. The order of the blocks was counterbalanced across the subjects. The experiment ended with 3 min long closed- and open-eyes resting state measurements. In this paper, we presented the analysis of EEG activity monitored in the course of dominant (right) index finger tapping. Experiment control and stimuli generation was performed by a custom-made application developed in Visual Basic (Microsoft, Redmond, WA, US). The software is available upon request.

### Data Recording and Processing

EEG was collected by a 128-channel Biosemi Active Two system and Biosemi Actiview recording software (BioSemi B.V., Amsterdam, Netherlands) with 2048 Hz sampling rate, and by using reference electrodes placed at the standard locations (CMS and DRL). Electrode impedances were kept below 5 kΩ. Markers denoting the key presses and the cue events were recorded via the standard trigger port of the EEG equipment.

Data processing was performed using custom-made MATLAB (The MathWorks Inc., Natick, MA, USA) scripts and by applying different MATLAB toolboxes developed for EEG analysis. Pre-processing started with filtering the raw recordings. EEG data was high- and low-pass filtered by using 4th order Butterworth filters with 0.5 Hz and 70 Hz cut-off frequencies, respectively. Power line noise was eliminated by a 50 Hz notch filter with Q = 45 quality factor. Zero-phase filtering was realized with the filtfilt() MATLAB function. In the next step, bad channels were excluded based on visual inspection of filtered data.

Cleaning of EEG recordings was performed in a semi-automatic way using a multi-stage artefact elimination approach based on independent component analysis (ICA) (Delorme et al. 2007; Onton et al. 2006) implemented in the EEGLAB toolbox (Delorme and Makeig 2004). Detection and elimination of the ICA components (ICs) contaminated by artefacts were carried out through six stages (Weiss et al. 2016) after segmenting EEG data into 3 s long epochs and merging data of left- and right tapping conditions. The sequential steps of eliminating artefacts were briefly as follows: (1) semi-automatic detection and removal of segments contaminated by artefacts based on different EEG time-series properties; (2) first ICA on the remaining segments; (3) semi-automatic detection and removal of segments with artefacts based on different IC activation time-series properties; (4) second IC decomposition on the remaining segments; (5) detection of ICs with artefact that were obtained by the second decomposition; (6) elimination of the ICs contaminated by artefacts by subtracting ICs with artefact from filtered recordings. ICs with artefact were detected on finger tapping-related EEG segments and based on visual inspection of ICs’ temporal, spatial and spectral properties, and by taking into account the results of MARA (Winkler et al. 2011), FASTER (Nolan et al. 2010) and ADJUST (Mognon et al. 2011) EEGLAB plug-ins. The base of the subjective decision was the number of the suggested rejections of these three automatic methods and the shape of the frequency spectral curve of the component (the brain activity has power law distribution or rises in the typical EEG frequencies, i.e., delta, theta, alpha, and beta). Occasionally registered bad electrodes were interpolated after artefact elimination using a fast 2D spatial spline interpolation implemented in the EEGLAB toolbox. The filtered continuous finger tapping EEG data (after removing the artefact ICs) were segmented to [−4500 to 2500] ms epochs synchronized with key press onset times as trigger events. In these trials, a final semi-automatic artefact elimination procedure (see step 1 above) was applied. At the end 110 trials (SD = 14) remained on average for each subject.

The cleaned EEG was down-sampled to 256 Hz. To reduce the effects of volume conduction, scalp current density transformation (SCD) was applied using the spherical spline Laplacian method (Perrin et al. 1989). SCD transformed data can be considered reference independent, the transformation enhances local activity and suppresses activity with broader spatial extent. Accordingly, SCD transformation can be used to reduce the volume conduction effect. Nevertheless, it does not replace the solution of the inverse problem and thus the obtained results can be interpreted in sensor space only. The SCD transform was performed using the CSD Toolbox (Kayser and Tenke 2006) with default parameters (unit sphere radius; the maximum degree of Legendre polynomials: 10; spline flexibility: m = 4; smoothing constant: λ = 10^-5).

The real source localization changes the dimension of the channels to source values. This step increases the data that have to be analysed to such an extent that it is untreatable after the time-frequency decomposition. With the usual solution of this problem (i.e., the examination the averaged values of some selected regions of interests only) it is hardly possible to delineate the topolographic extension of the synchronizations which is the aim of this study. Thus, the following analysis focused on the SCD transformed channel space.

To assess the spatial–temporal dynamics of brain oscillations, time–frequency decomposition of SCD data was performed by continuous wavelet transformation (Kronland-Martinet et al. 1987). For this purpose complex Morlet wavelets were applied (MATLAB cwt function with the ‘cmor1-1’ setting) in the [0.32 73] Hz frequency range with a logarithmic step ratio (f(n)/f(n-1) = 1.1). Event-related spectral perturbation (ERSP) was obtained by averaging baseline-corrected and log-transformed single-trial power values. Single-trial baseline correction was performed for all channels and frequencies separately according to the gain model (Grandchamp and Delorme 2011), i.e., all sample values were divided by the average power of baseline activity located in the [−3500 to −3000] ms time interval before the keypress onset time.

### Statistical Analysis

The behaviour of the participants was assessed by statistical evaluation of sensorimotor synchronization error and keypress duration. Synchronization error was defined as the time interval between the onset of keypresses and response stimuli, while keypress duration was calculated as the difference between keypress onset and corresponding offset times.

The finger tapping-related dynamics of brain oscillations were computed by comparing their power features to their baseline activity in the time interval between −3500 and −3000 ms before the onset of key presses. Statistical evaluation of EEG data was accomplished using cluster-based permutation testing (Maris and Oostenveld 2007) as implemented in the FieldTrip MATLAB toolbox (Onton et al. 2006). Cluster-based permutation testing is an efficient way for correcting the multiple-comparisons problem since it takes into account the adjacency of assessed samples in spatial–temporal-spectral domains. Clusters were formed on the basis of the adjacency of thresholded sample-level t-values. Thresholding of t-values and accordingly the complete statistical evaluation was performed by two different clusteralpha (CA) values. The CA1 = 0.001 threshold value was selected to minimize the occurrence of false positives (Eklund et al. 2016) and to reveal the boundaries of strongest effects; while CA2 = 0.05 was used to reveal potential false negative results, to validate significant CA1 clusters and evaluate their broader extent in the spatial–temporal-spectral domain. Spatial adjacency was determined by combining triangulation and distance methods implemented in the ft_prepare_neighbours() FieldTrip function (Onton et al. 2006). Distance threshold was set to 0.4. Cluster-level statistics were obtained by summing up the t-values corresponding to samples that form the clusters, and the maximum of cluster-level statistics was taken afterwards. Statistical significance of clusters was estimated based on 1000 Monte Carlo randomizations using 0.05 significance level.

Cluster-based statistical analysis might suppress smaller significant clusters beside stronger and larger clusters. To avoid this effect, the comparison against the baseline was calculated separately between 0.5 and 8 Hz and between 8 and 70 Hz. The analysis in the 8–70 Hz frequency range was divided to two time intervals (between −2700 and 450 ms and between 450 and 1200 ms) based on previous findings (Pfurtscheller and Lopes da Silva 1999) and visual inspection of spatial–temporal ERSP properties.

Statistical separation of delta and theta synchronization was executed on the normalized ERSP data to reveal the differences in their topography on the scalp. The normalization was computed in all frequency bins with the z-score function of the MATLAB. These data were averaged on the delta and on the theta frequency range in all point in the time-channel domain. The comparison between these two datasets was achieved with cluster-based permutation testing between −250 and 250 ms and on all channels using both 0.05 and 0.001 cluster alpha value.

To control the results of the cluster based statistics, data were processed with the classical statistics on three groups of channels on the contralateral (left) hemisphere (left frontocentral: C22-25/left central: D14, D18-21/left parietal: A6-7, D29 in the standard Biosemi 128 channel layout terminology). Selection of channels for calculation based on the baseline related comparison. T test was counted between the averaged ERSP values on the examined channel group and on the frequency range of delta and theta in all the study subjects.

## Results

The mean (± SD) duration of keypresses was: 223  ±  66 ms. The mean synchronization error was negative (−265  ±  175 ms), indicating that subjects pressed the key consequently before the visual cues.

**Fig. 1:**
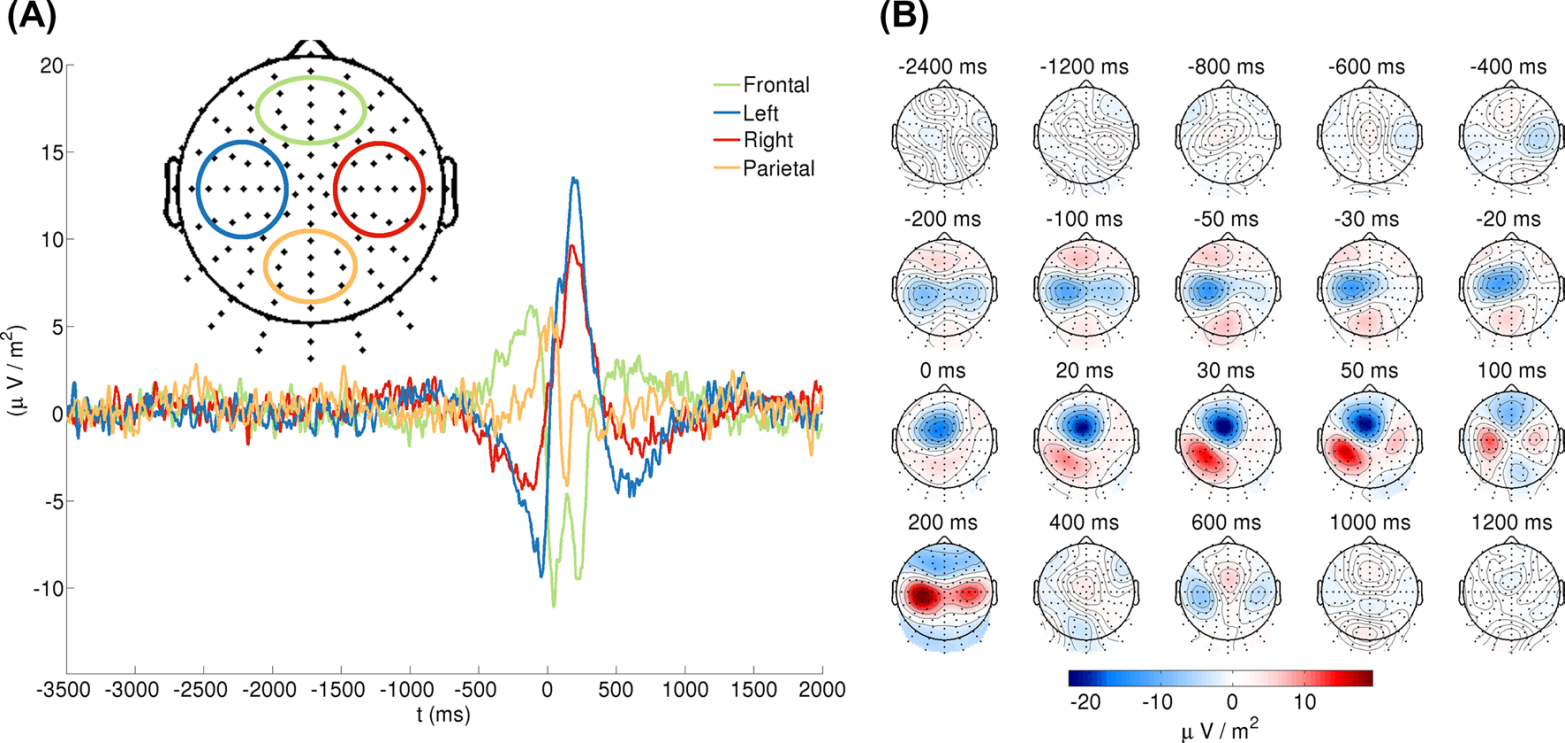
Grand average scalp current density (SCD) activity of the EEG related to right-hand (dominant hand) finger tapping. Panel **A** shows the time course of SCD activity for channel clusters that cover four topographic regions: left (contralateral) and right (ipsilateral) central as well as frontal and parietal regions. EEG activity was averaged across the electrodes that belong to particular channel clusters (see the figure inset). Zero on the horizontal axis denotes the onset of keypresses. Panel **B** shows the topography of SCD transformed EEG activity before, during and after the onset of finger tapping at specific time points of particular interest. EEG was averaged for ± 5 ms around the time points indicated above the maps

The distribution of the finger tapping-related scalp current density (SCD) on the scalp revealed a complex spatio-temporal pattern of brain activity (Fig. 1). A negative SCD trend can be observed in both the ipsilateral and contralateral central channels before the onset of key presses. In contrary to the central region, a positive trend is present in the frontal and parietal channels in the same time range. A flip of SCD polarity occurs around the onset of key presses within the first 50 ms after key presses. A fronto-central negativity that is slightly lateralized to the contralateral side is accompanied with a positive SCD pattern in the contralateral parietal and central-parietal channels. Afterwards, bilateral positivity with contralateral preponderance is present centrally, while negative SCD can be observed in the parietal and fronto-central regions between 50 and 300 ms. The polarity of this topography flips around 600 ms, and EEG activity drops to the baseline level about 1 s after the tapping.

**Fig. 2:**
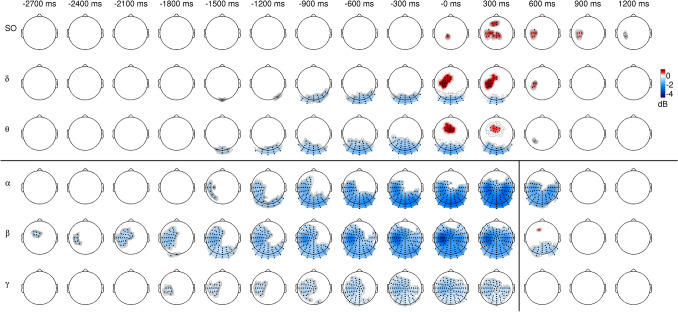
Spatio-temporal distribution of spectral perturbations (ERSP) related to right (dominant) hand finger tapping. ERSP was compared to the baseline activity from −3500 to 3000 ms with cluster-based statistical analysis using the 0.001 cluster alpha (CA1) value. Topographic maps stand for ERSP values averaged in 300 ms long time intervals (± 150 ms around the time points indicated above the maps) and specific frequency bands (SO – slow oscillations: 0.5–1 Hz, delta: 1–4 Hz, theta: 4–8 Hz, alpha: 8–15 Hz, beta: 15–30 Hz, gamma: 30–70 Hz). Only samples belonging to significant (p < 0.05) clusters were taken into consideration (red: positive, indicating significant event-related synchronization (ERS), blue: negative, indicating significant event-related desynchronization (ERD) and only electrodes belonging to these clusters were marked with black dots. Based on a priori information, the statistical evaluation was carried out separately for frequency ranges between 0.5 and 8 Hz and between 8 and 70 Hz (denoted by the black horizontal line). Furthermore, the frequency range from 8 to 70 Hz was divided into two separate time intervals (from -2700 to 450 ms and from 450 to 1200 ms, as marked by the black vertical line)

**Fig. 3:**
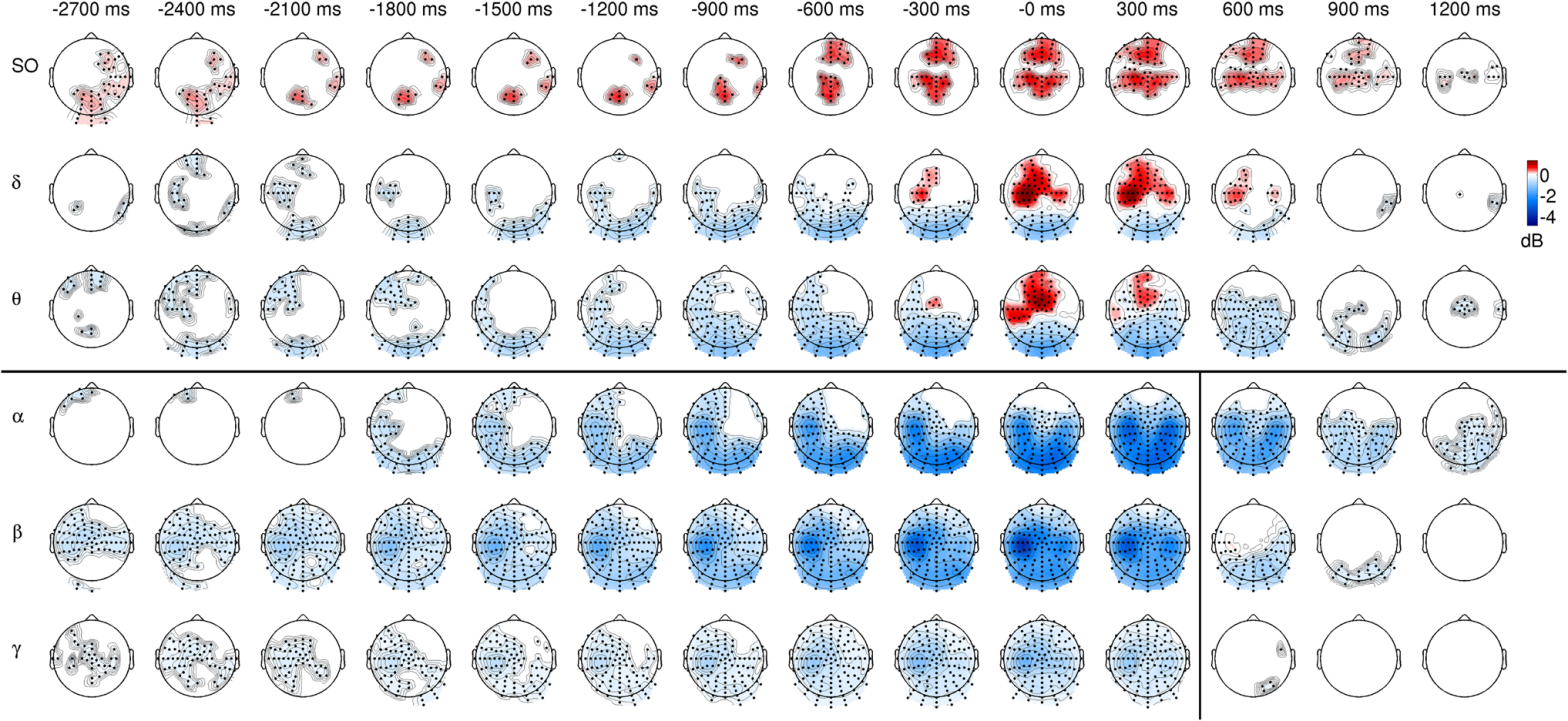
Spatio-temporal distribution of spectral perturbations (ERSP) related to right (dominant) hand finger tapping. ERSP was compared to the baseline activity from −3500 to 3000 ms with cluster-based statistical analysis using the 0.05 cluster alpha (CA1) value. Topographic maps stand for ERSP values averaged in 300 ms long time intervals (± 150 ms around the time points indicated above the maps) and specific frequency bands (SO – slow oscillations: 0.5–1 Hz, delta: 1–4 Hz, theta: 4–8 Hz, alpha: 8–15 Hz, beta: 15–30 Hz, gamma: 30–70 Hz). Only samples belonging to significant (p < 0.05) clusters were taken into consideration (red: positive, indicating significant event-related synchronization (ERS), blue: negative, indicating significant event-related desynchronization (ERD) and only electrodes belonging to these clusters were marked with black dots. Based on a priori information, the statistical evaluation was carried out separately for frequency ranges between 0.5 and 8 Hz and between 8 and 70 Hz (denoted by the black horizontal line). Furthermore, the frequency range from 8 to 70 Hz was divided into two separate time intervals (from -2700 to 450 ms and from 450 to 1200 ms, as marked by the black vertical line)

The comparison of power features to their baseline activity reveals the areas in the spatial–temporal-spectral domain where the estimated ERSP emerged from the general oscillatory activity. Significant ERS was found between 0.5 and 8 Hz with both CA levels (p = 0.001 and p = 0.05, see Figs. 2 and 3). The positive clusters include slow oscillations (SO, between 0.5 and 1 Hz) in channels above frontal, fronto-central, central and parietal areas as well as delta and theta oscillations in fronto-central and central regions with a contralateral preponderance. ERS of slow oscillations is present from the execution to 1200 ms with the CA = 0.001 (Fig. 2) and this interval is expanded to the entire examined time range (between −2700 and 1200 ms) with CA = 0.05 (Fig. 3). Statistical analysis with CA = 0.05, but not with the CA = 0.001, revealed frontal and occipito-parietal slow oscillation ERS in the early preparatory time (between −2700 and −300 ms; Fig. 3).

**Fig. 4:**
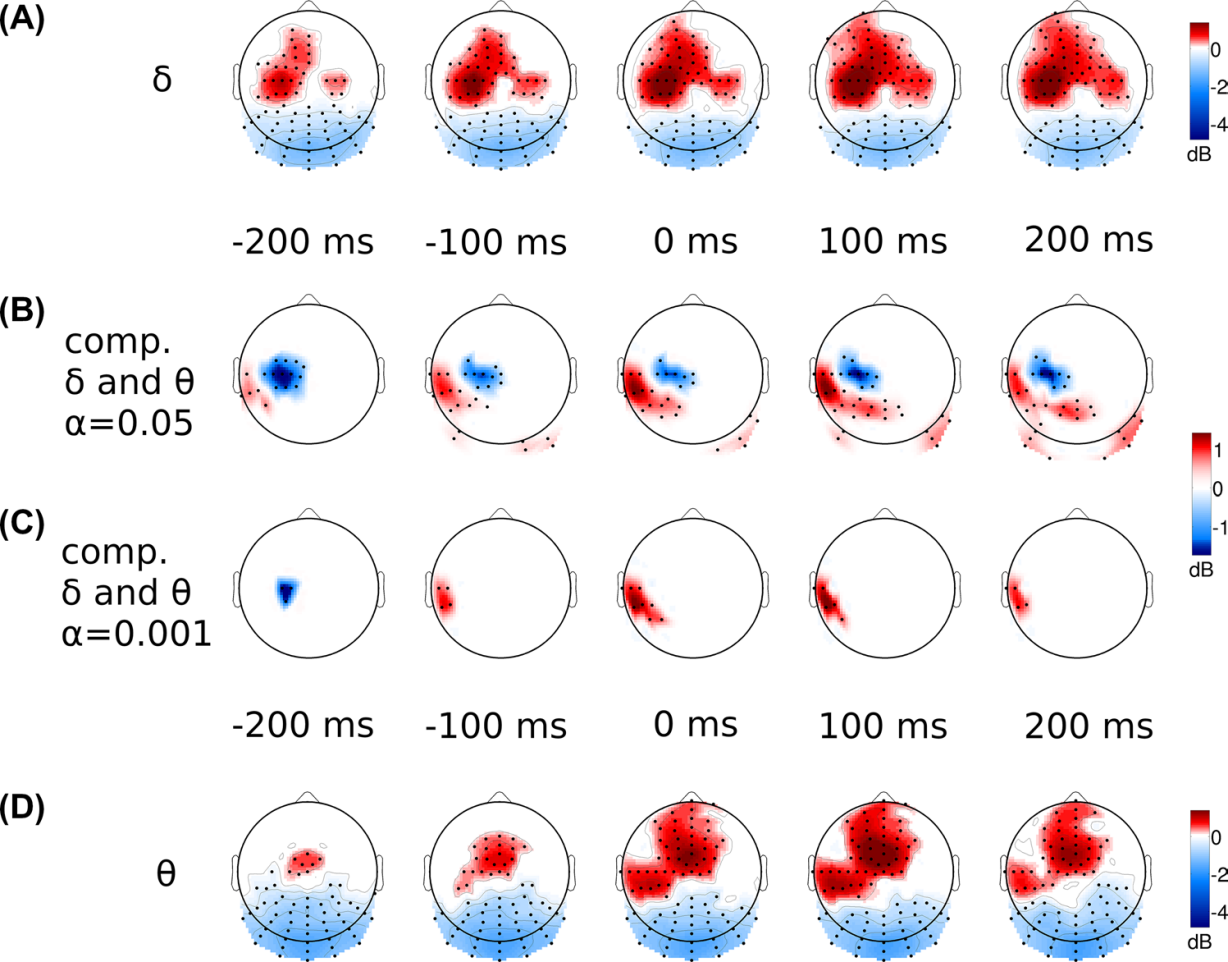
Comparison of the normalized finger tapping-related spectral perturbations (nERSP) in delta (1–4 Hz) and theta (4–8 Hz) frequency bands around the onset key presses (**B** and **C**) and the spectral perturbations (ERSP) in delta (**A**) and theta (**D**) frequency bands. The normalizations were achieved separately in all frequency bins to equalize the gross differences between the ERSP magnitudes and to focus only on the topographic differences. The nERSP was averaged in the delta and theta frequencies and was compared between these two range with cluster-based permutation testing using the both 0.05 (**B**) and 0.001 (**C**) cluster alpha value (between −250 and 250 ms). The upper (**A**) and lower (**D**) rows show the topographic distributions of the ERSPs (before the normalizations) in these two frequency ranges. Topographic maps stand for averaged nERSP or ERSP values in 100 ms long time intervals (± 50 ms around the key press time point) and the two frequency bands (delta: 1–4 Hz, theta: 4–8 Hz). Only samples belonging to significant clusters were taken into consideration with colourscale (red: positive, blue: negative) and only in these clusters were marked the symbolized electrodes with black dots. The colour scale shows the grand average values of the ERSP (in **A** and **D**) or the differences in the nERSP (in **B** and **C**) in the clusters (in the corresponding time and frequency interval), and all the area outside the clusters is painted to white. In the delta and theta comparison pictures (**B** and **C**) the positive (red) clusters represent the significantly higher nERSP values in the theta range while the negative (blue) clusters refer to the areas where the delta nERSP is higher significantly

Delta and theta ERS were significant in the time window from 0 to 300 ms with CA = 0.001 (Fig. 2) and from −300 to 600 ms with CA = 0.05 (Fig. 3). Considering the spatial distribution of significant effects within the positive cluster of larger extent (Fig. 3), differences between the topography of delta and theta ERS was observed around the execution of finger tappings. The delta waves appeared in the central contralateral area, while the theta part of the cluster is localized on the fronto-central and on the contralateral side in the central-parietal, parietal regions (see Fig. 4 for enlarged delta and theta effects around the execution of finger tappings).

In the frequencies between 8 and 70 Hz a significant negative cluster of ERD (p = 0.001) was found between -2700 ms and 900 ms by both CA which expands to the theta and delta range in the occipital channels at the time of the execution (Figs. 2 and 3).This cluster reflects ERD in occipital, parietal and central areas with a contralateral central preponderance. Furthermore, significant beta rebound ERS (p = 0.033) was also found around 600 ms in the contralateral fronto-central regions with the CA = 0.001 level (Fig. 2). With CA = 0.05, however, there is no significant cluster in this area (Fig. 3).

The cluster-based statistical analysis between the delta and theta normalized ERSP activities confirmed the differences between the topographic distribution of these two frequency range with both CA (Fig. 4). With CA = 0.05, the statistics found a significant (p = 0.002) negative cluster on the contra- and ipsilateral and on the occipital channels. This means that normalized delta ERSP is higher in these regions. (The comparison with CA = 0.001 did not support this difference.) A significant positive cluster was found on the contralateral fronto-central channels with both CA (with CA = 0.05, p = 0.037 and with CA = 0.001, p = 0.016) which shows that the theta oscillation has higher normalized ERSP in this region (Fig. 4). No significant cluster was found on the parietal channels where the comparison to the baseline revealed the parietal part of the theta synchronization.

**Fig. 5:**
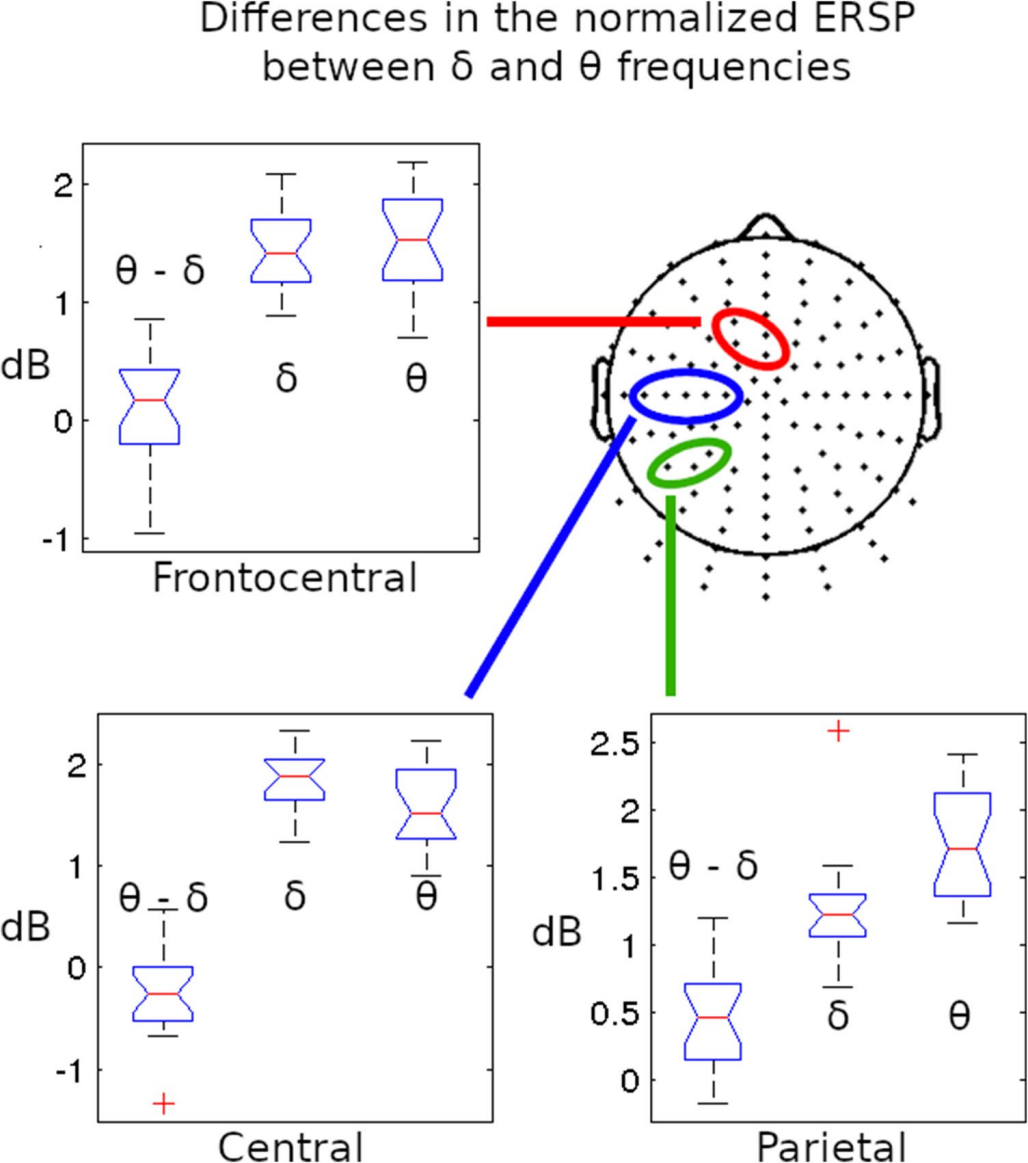
Differences in the normalized finger tapping-related spectral perturbations (nERSP) in delta (1–4 Hz) and theta (4–8 Hz) frequency bands around the onset key presses in three separated channel groups (left frontocentral: C22-25/left central: D14, D18-21/left parietal: A6-7, D29). The boxplots with δ and θ symbol represent the distribution of the average nERSP values in the delta and theta range (respectively) in the given channel groups. The boxplots with θ – δ symbols shows the spreads of differences between the averaged theta and delta nERSP values. The normalizations were achieved separately in all frequency bins to equalize the gross differences between the ERSP magnitudes and to focus only on the topographic differences

The classical t test gave same results as the cluster-based statistics. There was no significant difference between the delta and theta normalized ERSP in the parietal group of channels. The delta activity was significantly higher in the central group (p = 0.026) and in the fronto-central group the higher theta was proved (p < 0.0103). The distributions of the normalized ERSP values in these channel groups can be seen in the Fig. 5.

## Discussion

The aim of this study was to describe the movement-related delta and theta synchronizations with a detailed topographic distribution. Oscillatory activity in the frequency range from 0.5 to 70 Hz was assessed to confirm previous findings by using an experimental paradigm where movement execution was guided by visual attention, and to reveal the spatio-temporal relations of the delta and theta waves to the other oscillations. While we confirmed the classical movement-related alpha and beta desynchronization (ERD) (Pfurtscheller and Berghold 1989; Stancák and Pfurtscheller 1995) and beta rebound ERS (Pfurtscheller and Lopes da Silva 1999) phenomena, we could also separate the topographic location of delta and theta event related synchronization (ERS) in sensor space in the time of finger tapping execution. Another significant finding of the study was the double pattern of theta ERS in the parietal and fronto-central EEG channels.

In our experimental paradigm the initiation of movements has been coupled with a continuous visual stimulus where the expected key press time is predictable. Based on the literature of sensorimotor synchronization (Repp 2005; Repp and Su 2013) this condition could explain the revealed negative synchronization error.

The cluster based statistical analysis in the whole investigated range of the spatial–temporal-spectral domain yields more clusters where the oscillatory power significantly differs from the baseline activity. The alpha, beta and gamma ERD, the largest cluster, encompasses the occipital, parietal and the central lateral areas which refer to visual processing (Koshino and Niedermeyer 1975; Pfurtscheller et al. 1994) and the sensorimotor processing (Pfurtscheller and Aranibar 1979; Pineda 2005).

The movement-related beta ERD is generally followed by rebound oscillations (ERS) that might reflect the activity of supplementary and primary motor brain areas (Neuper and Pfurtscheller 1996; Pfurtscheller et al. 1996b). The contralateral-central beta ERS found after the keypress around 600 ms in this study is in accordance with previous observations. However, we could not confirm the alpha synchronization in the contralateral central channels shown by other groups (Pfurtscheller et al. 1996a; Pineda 2005).

The earlier description of the movement-related delta and theta oscillations was not described separately (Kirmizi-Alsan et al. 2006; Yamanaka and Yamamoto 2010), and it is assumed that these effects originate from contralateral primary motor (Popovych et al. 2016) or supplementary motor areas (Popivanov et al. 1999). If we consider the delta and theta synchronization as correlates of different functions of motor activity and suppose that these are originated from different brain regions this disambiguity can be solved. Using invasive recordings, delta oscillations in the human primary motor cortex are shown to entrain to the interval of informative cues used for prompting of movement planning (Saleh et al. 2010), while the origin of the theta oscillations assumed from medial frontal, the supplementary motor areas (Pellegrino et al. 2018; Schramm et al. 2019), and medial frontal theta activity may represent communication between the frontal midline and other brain areas during cognitive control (Cavanagh and Frank 2014). Thus, our results about the separated delta and theta oscillations which are both related to the movement execution shed new light on these previous speculations.

## Limitations and Future Directions

The SCD transformed data in the channel space are suitable to delineate the topographic extension of the synchronizations in the delta and theta frequency range. It is hard, however, to determinate the cortical sources of these activities besides the described topographic distribution on the scalp. The overview of the literature might help to make an estimation only regarding the sources of some channel regions in special frequency ranges. A further step could be source localization with individual brain models (as the average brain model with general electrode setting is not sufficient), and the application of neuronavigation methods.

Another limitation of the recent study is the predictable periodicity of the press condition. This could affect movement initiation as participants might anticipate the timing of key presses by implicit perception of the timing or rhythm. As it has been mentioned above, our aim with this specific paradigm was to model the everyday situation in which voluntary movement is involved as a response to a not totally unpredictable visual stimulus. While the periodicity of the stimuli has its downsides, this is the only way to make the stimuli predictable. Most importantly, this limitation of the paradigm does not affect the conclusion of this study.

## Conclusions

Delta and theta oscillations are not only integrated parts of the movement related cortical activations but show different topographic distributions on the scalp as well.

## Data Availability

The data that support the findings of this study are available from the corresponding author upon reasonable request.
